# *Listeria monocytogenes*-infected human monocytic derived dendritic cells activate Vγ9Vδ2 T cells independently of HMBPP production

**DOI:** 10.1038/s41598-021-95908-5

**Published:** 2021-08-11

**Authors:** Alejandro F. Alice, Gwen Kramer, Shelly Bambina, Keith S. Bahjat, Michael J. Gough, Marka R. Crittenden

**Affiliations:** 1 Robert W. Franz Cancer Center, Earle A. Chiles Research Institute, Providence Portland Medical Center, 4805 NE Glisan St, Portland, OR 97213 USA; 2 The Oregon Clinic, Portland, OR 97213 USA; 3 Present Address: Astellas Pharma US, 100 Kimball Way, South San Francisco, CA 94080 USA

**Keywords:** Infectious diseases, Pathogens

## Abstract

Gamma-delta (γδ) T cells express T cell receptors (TCR) that are preconfigured to recognize signs of pathogen infection. In primates, γδ T cells expressing the Vγ9Vδ2 TCR innately recognize (E)-4-hydroxy-3-methyl-but- 2-enyl pyrophosphate (HMBPP), a product of the 2-C-methyl-D-erythritol 4- phosphate (MEP) pathway in bacteria that is presented in infected cells via interaction with members of the B7 family of costimulatory molecules butyrophilin (BTN) 3A1 and BTN2A1. In humans, *Listeria monocytogenes* (*Lm*) vaccine platforms have the potential to generate potent Vγ9Vδ2 T cell recognition. To evaluate the activation of Vγ9Vδ2 T cells by *Lm*-infected human monocyte-derived dendritic cells (Mo-DC) we engineered *Lm* strains that lack components of the MEP pathway. Direct infection of Mo-DC with these bacteria were unchanged in their ability to activate CD107a expression in Vγ9Vδ2 T cells despite an inability to synthesize HMBPP. Importantly, functional BTN3A1 was essential for this activation. Unexpectedly, we found that cytoplasmic entry of *Lm* into human dendritic cells resulted in upregulation of cholesterol metabolism in these cells, and the effect of pathway regulatory drugs suggest this occurs via increased synthesis of the alternative endogenous Vγ9Vδ2 ligand isoprenyl pyrophosphate (IPP) and/or its isomer dimethylallyl pyrophosphate (DMAPP). Thus, following direct infection, host pathways regulated by cytoplasmic entry of *Lm* can trigger Vγ9Vδ2 T cell recognition of infected cells without production of the unique bacterial ligand HMBPP.

## Introduction

The largest proportion of circulating human γδ T cells express Vγ9Vδ2 TCRs^1^. These TCRs share a characteristic Vγ9JγPCγ1 chain paired with a Vδ2 chain which respond in a TCR-dependent fashion to non-peptidic pyrophosphate compounds or phosphoantigens (p-Ag)^2,3^. These include the microbial derived compound (E)-4-hydroxy-3-methyl-but- 2-enyl pyrophosphate (HMBPP)^3–7^, which is generated in most members of *Eubacteria* by the alternative 2-C-methyl-D-erythritol 4- phosphate (MEP) pathway, and is a highly potent activator of Vγ9Vδ2 T cells^4,5,8–12^. Since this pathway is active in pathologically significant bacteria such as *Bordetella, Chlamydia, Clostridia, Escherichiae, Mycobacteria, Neisseriae, Salmonellae, Shigellae, Vibrios,* and *Yersiniae* as well as in protozoan such as *Plasmodium* species (reviewed in^2,6,13^), Vγ9Vδ2 T cells act as an innate T cell population pre-configured to recognize a wide array of bacterial infections though their metabolic activity. In addition to bacterial-derived HMBPP, host-cell-derived isoprenyl pyrophosphate (IPP) and its isomer dimethylallyl pyrophosphate (DMAPP) produced in eukaryotic cells through the classic mevalonate pathway can act as a p-Ag and stimulate Vγ9Vδ2 T cell responses, although this stimulation is less efficient than that obtained with HMBPP^6,9,14–16^.

Vγ9Vδ2-mediated p-Ag sensing requires cell–cell contact and depends on both Vγ and Vδ chains^17,18^. An essential prerequisite for p-Ag sensing is the expression of butyrophilin (BTN) 3A1 in the target cell^19^. Several studies provided evidence for p-Ag binding to the intracellular B30.2 domain of BTN3A1 and for a p-Ag induced conformational change of this protein^10,20,21^. More recently, butyrophilin 2A1 (BTN2A1) was identified as a key ligand that binds to the Vγ9 TCR γ-chain. BTN2A1 associates with BTN3A1 independently of p-Ag stimulation and act together to initiate responses to pAg^22,23^. Recent studies have implicated binding of a second ligand to a separate TCR domain incorporating Vδ2^22,23^, and additional family members regulate optimal presentation of phosphoantigens through BTN3A1^24^. Thus, the complete description of the p-Ag sensing by the γδ TCR is not yet fully understood.

*Listeria monocytogenes* (*Lm*) is a Gram-positive facultative intracellular pathogen. In mice, *Lm* infection generates strong *Lm* antigen-specific αβ T cell responses and the bacteria has been genetically manipulated to serve as a tumor antigen vaccine platform^25–28^, even in the face of host tolerance^29^. In mice, γδ T cells are mainly involved in the early phase of the immune response to Lm infection^30^, but due to multifaceted interactions with the host immune system *Lm* is a potent inducer of αβ CD8 T cell responses in mice^27^. While mice have proven to be an excellent model to study the dynamics of bacterial infection and host immune response, they lack both the critical butyrophilin family genes *BTN3A1* and *BTN2A1*, and the Vγ9Vδ2 T cells capable of recognizing HMBPP or IPP. Thus, murine models may not faithfully recapitulate events in humans experiencing the same infection. In contrast to mouse infections, *Lm* infection in humans generates a specific expansion of Vγ9Vδ2 T cells^31,32^, which is of unclear significance to the use of *Lm* as an αβ T cells vaccine platform. In addition, there are variations in HMBPP production in *Listeria* strains^33^, which has the potential to impact listeriosis in humans.

Synthesis of HMBPP via the MEP pathway in *Lm* is dependent on the gene *gcpE* (also referred to as *ispG*)^34^. Cellular extracts obtained from *Lm* wild type (wt) strains can induce the expression of CD69 and CD25 on Vγ9Vδ2 T cells in vitro, whereas extracts obtained from a HMBPP-negative *ΔgcpE* strain failed to do so^35^. Similarly, supernatants from neutrophils infected with *Listeria innocua* expressing the *gcpE* gene (HMBPP overproducer) were able to activate Vγ9Vδ2 T cells coincubated with monocytes^36^. Notably the monocyte population and the presence of HMBPP were essential for this activation^36–38^. Furthermore, non-human primates that were intranasally infected with a wt strain showed Vγ9Vδ2 T cells expansion that was diminished when a *ΔgcpE* strain was tested^39–41^. These data strongly support a model where *Lm* infection generates HMBPP which results in BTN3A1-mediated activation of Vγ9Vδ2 T cells.

The fact that bacterial extracts can activate Vγ9Vδ2 T cells in a HMBPP and BTN3A1-dependent manner may not accurately model in vivo infection, where cellular entry is critical. In this work we aimed to explore the effects of *Lm* entry into human dendritic cells and the effect of a HMBPP-negative *ΔgcpE* strains on Vγ9Vδ2 T cell activation. Surprisingly, we found that Lm-infected human monocyte-derived dendritic cells (Mo-DC) cells are able to activate Vγ9Vδ2 cells independently of HMBPP production. We found that during the first hours of infection of Mo-DC there is a change in the transcription of genes involved in cholesterol biosynthesis and export (*ABCA1* and *ApoA1*) that would result in an increase in the intracellular IPP pool. Changes in the levels of this metabolite during the infection affect the activation of the Vγ9Vδ2 T cells. In addition, the *CH25H* gene, a gene that usually is induced by Type I IFN in macrophages during *Lm* infection in mice, is not activated in Mo-DC leading to a lack of repression of the cholesterol synthesis. Importantly, these data demonstrate that during direct infection of human DC, Vγ9Vδ2 T cells can be activated independently of HMBPP production via regulation of host cholesterol biosynthesis.

## Results

### HMBPP-independent activation of Vγ9Vδ2 T cells

**Figure 1:**
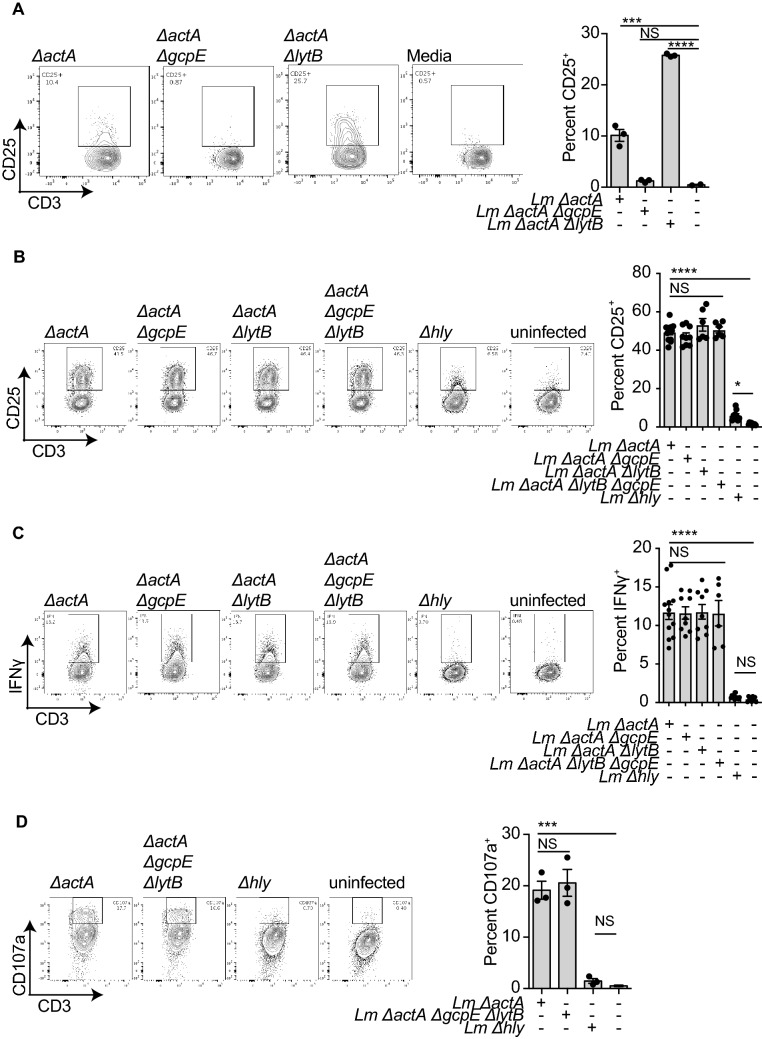
Vγ9Vδ2 T cell activation by bacterial cell extracts and *Lm*-infected Mo-DC. (**A**) Bacterial extracts from the indicated *Lm* strains were added to PMBC obtained from healthy donors. Cells were harvested at 72 h post-incubation, stained and analyzed by flow cytometry. Left panel, flow-cytometry plots and right panel, quantitation of percent CD25^+^ δ2^+^ CD3^+^ cells are representative results for at least 4 biologically independent experiments. (**B**,**C,D**) Mo-DC were infected with the indicated *Lm* strains at a MOI = 10, after 1 h cells were washed and ex-vivo expanded Vγ9Vδ2 T cells added. Cells were incubated for 18 h (IFNγ and CD25) or another 2 h in presence of monensin for CD107a staining. Cells were processed and stained for flow cytometry. Left panels, flow-cytometry plots and right panels, quantitation of percent IFNγ^+^ δ2^+^ CD3^+^, CD25^+^ δ2^+^ CD3^+^or CD107a^+^ δ2^+^ CD3^+^ cells are representative results of 4 to 6 independent experiments. Data represents the mean ± SEM of each group (n = 3–9). Statistics calculated by one-way ANOVA with Tukey’s correction; * = *p* < 0.05, *** = *p* < 0.001, **** = *p* < 0.0001, ns = no significant differences observed between the groups analyzed.

To study HMBPP-related Vγ9Vδ2 T cell activation we constructed a *ΔgcpE* version of the attenuated *ΔactA* strain that cannot synthesize HMBPP (Suppl Fig. 1A). ActA plays a major role in listerial virulence, and its absence leaves bacteria intracellularly immotile and essentially non-infectious. As a consequence, the *ΔactA* strain can escape the phagolysosome but is unable to infect surrounding cells. This attenuated strain has the same genetic background of several *Lm*-based cancer vaccine platforms. As a comparison, we also constructed a *ΔlytB* mutant strain that lacks the 4-Hydroxy-3-methylbut-2-enyl diphosphate reductase enzyme that metabolizes HMBPP (also referred to as *ispH*), and so results in increased HMBPP production. All the mutations were confirmed by PCR (Suppl Fig. 1B). To confirm published results^34^, we evaluated the ability of bacterial lysates of the *ΔactA*, *ΔactAΔgcpE,* and *ΔactAΔlytB* strains to activate Vγ9Vδ2 T cells in vitro. As expected, *ΔactA* extracts resulted an increase in the activation marker CD25 on Vγ9Vδ2 T cells, this was lost in extracts from the *ΔactAΔgcpE* strain,and increased in extracts from the *ΔactAΔlytB* (Fig. 1A). These data support the observation that HMBPP is the main factor in the activation of Vγ9Vδ2 T cells by *Listeria* cell extracts^34,35^.

To model Vγ9Vδ2 T cell activation during an active *Lm* infection, we directly infected human monocyte-derived DC (Mo-DC) at a range of MOI. Extracellular bacteria were removed by washing, then Vγ9Vδ2 T cells were added at a range of Mo-DC:T cell ratios. Activation of Vγ9Vδ2 T cells was determined by CD25 expression and IFNγ production using flow cytometry (Suppl Fig. 2). Surprisingly, we found that Vγ9Vδ2 T cells could be activated equally by *ΔactA* or the *ΔactAΔgcpE* strain (Fig. 1B), suggesting that in this setting Vγ9Vδ2 T cell activation was independent of HMBPP production. In support of this, the HMBPP overproducer *ΔactAΔlytB* strain also showed similar levels of Vγ9Vδ2 T cells activation to the *ΔactA* and the *ΔactAΔgcpE* strain (Fig. 1B). The response was independent of the MOI used in the experiments (Suppl Fig. 3A). To rule out the possibility that small quantities of HMBPP were still produced in the *ΔactAΔgcpE* strain, we constructed a double *ΔactAΔgcpEΔlytB* strain (Suppl Fig. 1A), which produced similar levels of activation to the control strain (Fig. 1B,C,D). Finally, to characterize the immediate activation response of Vγ9Vδ2 T cells we analyzed degranulation by measuring surface expression of CD107a, which confirmed equivalent activation in the absence of HMBPP (Fig. 1D).

To assess Vγ9Vδ2 T cell function, we assessed cytotoxic activity against infected Mo-DC. We found that the cytotoxicity of Vγ9Vδ2 T cells against *Lm*-infected Mo-DC is also not affected by the phosphoantigen production (Suppl Fig. 3B). This activity of Vγ9Vδ2 T cells was dependent on cytoplasmic entry of *Lm*, since Mo-DC infected with the *Δhly Lm* strain, which are unable to escape from the phagolysosome, were poorly able to activate Vγ9Vδ2 T cells despite functional HMBPP production and Vγ9Vδ2 T cells had poor cytotoxicity towards *Δhly*-infected Mo-DC (Fig. 1 and Suppl Fig. 3B ii,iii). We confirmed these data using naïve Vγ9Vδ2 T cells instead of *ex-vivo* expanded activated Vγ9Vδ2 T cells (Suppl Fig. 4A). Importantly, comparable results were obtained with Mo-DC and Vγ9Vδ2 T cells expanded from at least 4 different healthy donors (Suppl Fig. 4B). These results demonstrate that *Lm*-infected Mo-DC can activate Vγ9Vδ2 T cells and these cells, in turn, can kill *Lm*-infected cells independently of HMBPP production. However, cytoplasmic invasion of Mo-DC by *Lm* was essential for maximal cytotoxicity activity and Vγ9Vδ2 T cell activation. These data suggest that cytoplasmic entry of *Lm* results in an alternative pathway of Vγ9Vδ2 T cell activation independent of HMBPP production.

### Mechanism of γδ T cell recognition

**Figure 2:**
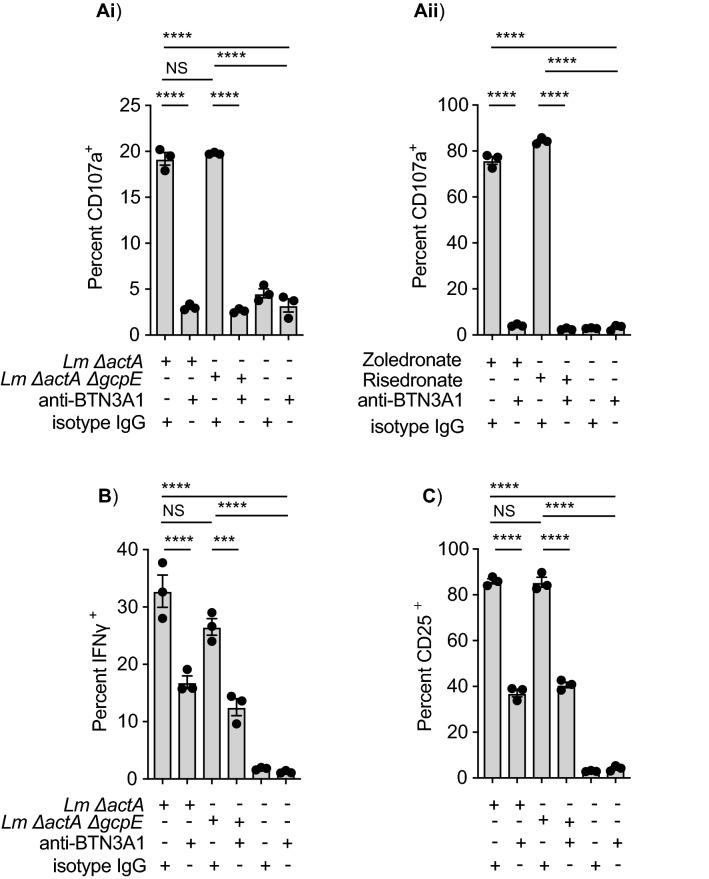
The role of BTN3A1 in Vγ9Vδ2 T cell activation by *Lm*-infected Mo-DC. (**A**) Human Mo-DCs were infected with the indicated *Lm* strains at a MOI = 10 or left uninfected. After 1 h of incubation at 37 °C, cells were washed twice to remove extracellular bacteria and 100 μl of fresh media including 50 μg/ml gentamycin and either 10 μg/ml anti-BTN3A1 blocking antibody or 10 μg/ml IgG isotype were added. After 45 min of further incubation at 37C, purified ex-vivo expanded Vγ9Vδ2 T cells were added to each well. Cells were incubated another 2 h in presence of monensin for CD107a staining. **i**) Quantitation of percent CD107a^+^ δ2^+^ CD3. **ii**) Mo-DC cells were pre-incubated with zoledronate (50 μM) or risedronate (50 μM), washed and further incubated with either anti-BTN3A1 blocking antibody or IgG isotype. Ex-vivo expanded Vγ9Vδ2 T cells were added to each well and CD107a quantitation performed as above. (**B**) Quantitation of percent IFNγ^+^ δ2^+^ CD3 and **C**) percent CD25 δ2^+^ CD3. Cells were processed and stained for flow cytometry as described in “Methods” section. Graphs are representative results of 2 to 4 independent experiments. Data represents the mean ± SEM of each group (n = 3). Statistics calculated by one-way ANOVA with Tukey’s correction; *** = *p* < 0.001, **** = *p* < 0.0001, ns = no significant differences observed between the groups analyzed.

BTN3A1 has been implicated in Vγ9Vδ2 T cell activation by phosphoantigen binding to its intracellular 30.1 domain^8,19^. To determine whether BTN3A1 was necessary for Vγ9Vδ2 T cells activation during *Lm* infection, we used the anti-BTN3A1 blocking monoclonal antibody (mAb) clone 103.2 that has been previously described to be able to inhibit BTN3A1-phosphoantigen induced Vγ9Vδ2 T cells activation^19^. Compared to IgG control-treated *Lm*-infected Mo-DC, 103.2 mAb treated Mo-DC cells infected with either HMBPP-positive or -negative *Lm* strains were unable to activate the CD107a expression in Vγ9Vδ2 T cell (Fig. 2 Ai). Control experiments where zoledronate- or risedronate-pulsed Mo-DC were incubated with Vγ9Vδ2 T cells in the presence or absence of the anti-BTN3A1 antibody showed similar effect in the CD107a expression in the T cells confirming the specific inhibition due to this antibody (Fig. 2 Aii). These results demonstrate that BTN3A1 is needed to fully activate these T cells when exposed to *Lm*-infected Mo-DC. They also suggest that while HMBPP is not essential to activate Vγ9Vδ2 T cells, BTN3A1 remains important and highlights a potential role of other BTN3A1-binding phosphoantigens in Vγ9Vδ2 T cell activation following direct infection.

We also analyzed the expression of the later activation markers CD25 and IFNγ in Vγ9Vδ2 T cells following BTN3A1 blockade. Vγ9Vδ2 T cell incubated with *Lm*-infected Mo-DC resulted in Vγ9Vδ2 T cell activation as measured by CD25 and IFNγ expression, which was significantly reduced by BTN3A1 antibody blockade. However, this inhibition was not as complete as that observed for CD107a in the short-term assay (Fig. 2B,C). While these studies confirm that expression of CD25 and IFNγ in Vγ9Vδ2 T cell exposed to *Lm*-infected Mo-DC is independent of the HMBPP, their expression in Vγ9Vδ2 T cells appears to be partially independent of phosphoantigen-BTN3A1 presentation. This BTN3A1-independent activation may correspond with cytokines induced in these cells following *Lm*-infection, as has previously been described^42,43^. Other co-stimulatory molecules such as NKG2D have been shown to activate Vγ9Vδ2 T cells^6,44–46^. To evaluate the role of NKG2D, we evaluated the effect of an anti-NKG2D blocking antibody on Vγ9Vδ2 T cell activation by *Lm*-infected Mo-DC. Neither cytotoxicity towards *Lm*-infected Mo-DC, CD107a translocation, nor IFNγ production by these Vγ9Vδ2 T cells were affected by the anti-NKG2D antibody (Suppl Fig. 5A).

Together these results indicate that *Lm*-infected Mo-DC are able to activate the degranulation of Vγ9Vδ2 T cell through a phosphoantigen-BTN3A1 interaction with the Vγ9Vδ2 TCR independently of HMBPP expression, but later CD25 activation and IFNγ production is partially independent of BTN3A1 interactions.

### Role of endogenous IPP synthesis

**Figure 3:**
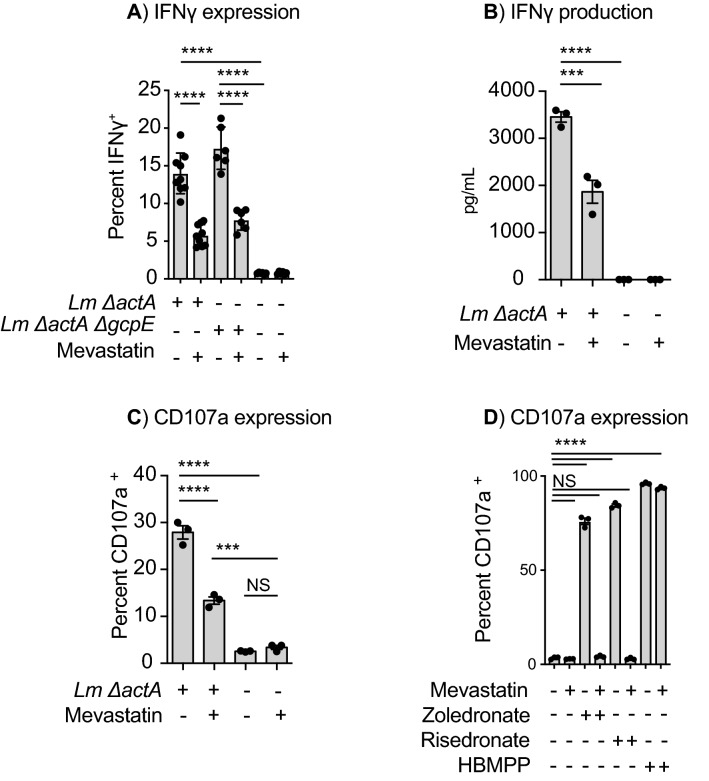
Effects of mevastatin in Vγ9Vδ2 T cell activation by *Lm*-infected Mo-DC. Mo-DC were incubated with mevastatin, infected or left uninfected with the indicated *Lm* strain, washed then incubated with ex-vivo expanded Vγ9Vδ2 T cells. (**A**) Cells were stained and quantitation of percent IFNγ^+^ δ2^+^ CD3^+^ analyzed for flow cytometry, (**B**) IFNγ was quantified in the supernatants by Luminex. (**C,D**) Uninfected and *Lm*-infected Mo-DC were treated as described, washed, incubated with Vγ9Vδ2 T cells in the presence of an anti-CD107a antibody and monensin and percent CD107a^+^ δ2^+^ CD3^+^ analyzed for flow cytometry. Graphs are representative results of 2 to 5 independent experiments. Data represents the mean ± SEM of each group (n = 3–6). Statistics calculated by one-way ANOVA with Tukey’s correction; *** = *p* < 0.001, **** = *p* < 0.0001, ns = no significant differences observed between the groups analyzed.

While HMBPP is a potent bacterial-derived ligand of Vγ9Vδ2 T cells, endogenous IPP is also able to activate Vγ9Vδ2 T cells, but at a lower affinity^47^. Therefore, we explored whether changes in the internal IPP pool of Mo-DC cells is responsible for Vγ9Vδ2 T cell activation following infection. To evaluate the role of endogenous mevalonate pathways and IPP synthesis in Vγ9Vδ2 T cells activation by *Lm*-infected Mo-DC, we incubated Mo-DC cells with statins during infection with *Lm*. Infected cells were then washed and exposed to Vγ9Vδ2 T cells to evaluate their activation. We observed that statin treatment decreased IFNγ production and CD107a expression by Vγ9Vδ2 T cells exposed to *Lm-*infected Mo-DC (Fig. 3A–C), suggesting a potential role for endogenous IPP production in Vγ9Vδ2 T cell activation following direct infection. In agreement with these data, zoledronate and risedronate treatments increased Vγ9Vδ2 T cell activation that was blunted in the presence of mevastatin while HMBPP activation was not affected by the presence of the statin (Fig. 3D). Importantly, the Vγ9Vδ2 T cells activation by the *ΔactAΔgcpE* strain was also sensitive to statin treatment (Fig. 3A). The effect of statins did not appear to be due to effects on *Lm* infection of the Mo-DC, as has been recently described for murine bone marrow-derived macrophages^48^, since statin treatment did not significantly affect *Lm* proliferation in Mo-DC (Suppl Fig. 5C).

**Figure 4:**
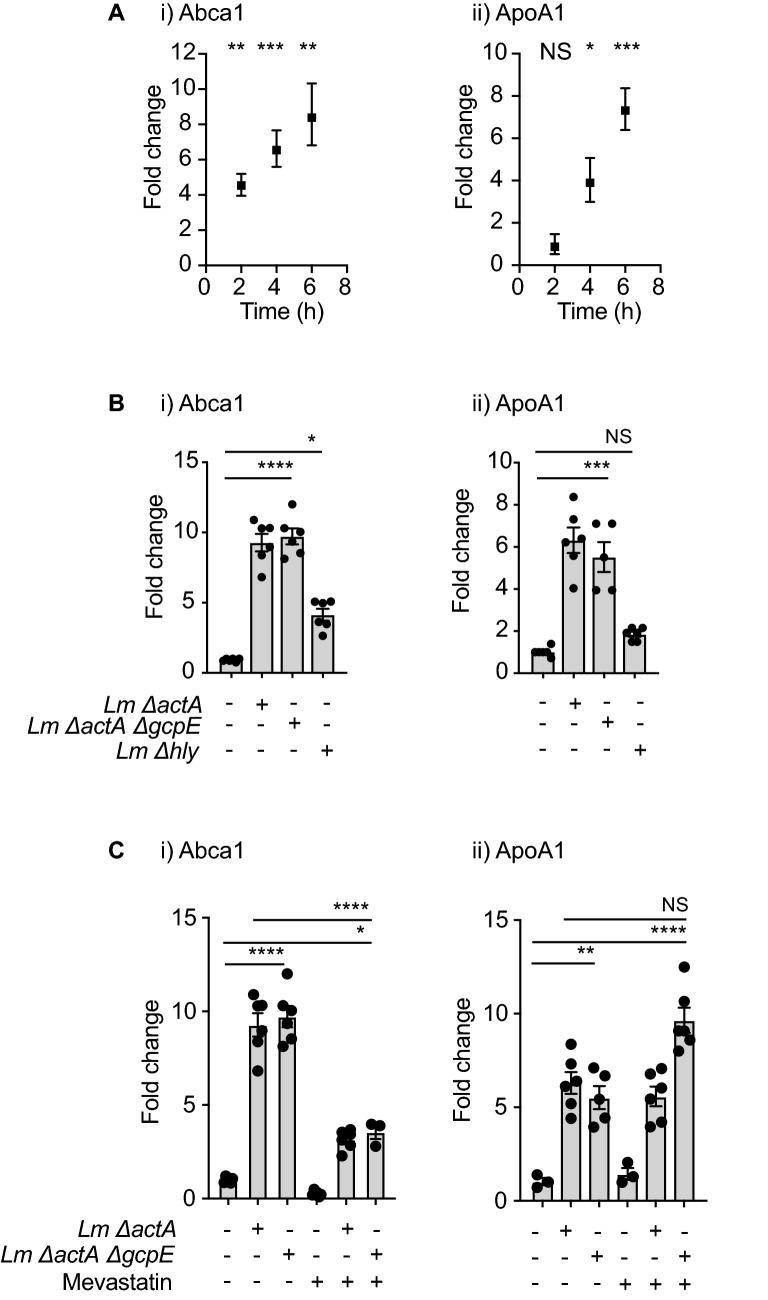
*ABCA1* and *APOA1* gene expression changes during *Lm* infection of Mo-DC. (**A**) Mo-DC were infected as described, at the indicated time points cells were harvested and RNA extracted for quantitative real time PCR analysis of gene expression. (**i)** *ABCA1* and (**ii**) *APOA1* gene expression at various time points after *ΔactA* infection; (**B**) (**i**) *ABCA1* and (**ii**) *APOA1* gene expression in Mo-DC at 6 h post infection (hpi) with the indicated *Lm* strains. (**C**) (**i**) *ABCA1* and (**ii**) *APOA1* gene expression in uninfected or *Lm*-infected Mo-DC treated or untreated with mevastatin. Fold changes are expressed as 2^-ΔΔCt^, where the internal control is the *Gapdh* gene and the control samples are uninfected cells. Results are representative of 2 to 5 biologically independent experiments. Data represents the mean ± SEM of each group (3–6). Statistics calculated by unpaired *t* test (**Ai** and **Aii**) or one-way ANOVA with Tukey’s correction (**Bi**, **Bii**, **Ci** and **Cii**); * = *p* < 0.05, ** = *p* < 0.01, *** = *p* < 0.001, **** = *p* < 0.0001, ns = no significant differences observed between the groups analyzed.

**Figure 5:**
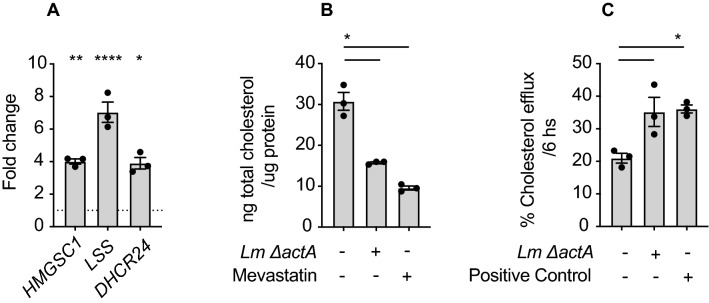
Changes in cholesterol metabolism in *Lm*-infected Mo-DC. (**A**) Changes in the gene expression of the indicated genes in *Lm*-infected Mo-DC after 6 hpi. Fold changes were expressed as 2^-ΔΔCt^, where the internal control is the *Gapdh* gene and the control samples are uninfected cells. Results obtained for some of the genes are representative of at least 4 biologically independent experiments. (**B**) Cholesterol content and (**C**) cholesterol efflux from uninfected or *Lm*-infected Mo-DC were quantified at 6 hpi as described in “Methods ” section. Results shown are representative of 2 biologically independent experiments. Data represents the mean ± SEM of each group (n = 3). Statistics calculated by unpaired *t* test (**A**) or one-way ANOVA with Tukey’s correction (**B,C**); * = *p* < 0.05, ** = *p* < 0.01, **** = *p* < 0.0001.

Recently, Castella et al.^49^ demonstrated that zoledronic acid and statins regulate the expression of the ATP-binding cassette transporter A1 (ABCA1) and apolipoprotein A1 (ApoA1) encoding genes in Mo-DC. ABCA1 is a well-studied major cholesterol exporter and ApoA1 is a component of the high-density lipoprotein (HDL), which is the molecule that associates to the ABCA1 exporter and transports cholesterol and phospholipids into the bloodstream. These authors propose that increasing concentrations of intracellular IPP leads to an increase in the expression of ABCA1 and ApoA1 and as a consequence these two proteins interact with BTN3A1, export of IPP to the extracellular space, and Vγ9Vδ2 T cell activation. We identified that both *ABCA1* and *APOA1* genes are induced in the first 2–6 h of Mo-DC infection (Fig. 4A), and still occurs following infection with the *ΔactAΔgcpE* strain (Fig. 4B). At these same time points we did not detect transcriptional regulation of *BTN3A1* in infected Mo-DC (not shown). Importantly, we did not detect induction of the *APOA1* gene and only a modest induction of the *ABCA1* gene following infection with the *Δhly* strain, suggesting that cytosol invasion was essential to augment the expression of these two genes (Fig. 4B). To determine whether *Lm* induction of the *ABCA*1 gene and *APOA1* was dependent on phosphoantigen production in the cells we blocked production with mevastatin in conjunction with infection and determined gene induction. Mevastatin treatment decreased transcription of *ABCA1*, and mevastatin limits, but does not completely prevent *ABCA1* expression following infection with *Lm* (Fig. 4C). In Mo-DC, the expression of the *APOA1* gene was not affected by the statins and induction of *APOA1* following *Lm* infection was unaltered by statins (Fig. 4C). To perform a more extensive analysis of the genes involved in the mevalonate/cholesterol and isoprenoid metabolism in the host cell, we profiled these genes at early time points following *Lm* infection of Mo-DC. We identified an increase in the transcription of *LSS* (lanosterol synthase), *HMGCS1* (3-Hydroxy-3-Methylglutaryl-CoA Synthase 1) and *DHCR24* (24-Dehydrocholesterol Reductase) (Fig. 5A), suggesting increased activity of the mevalonate/cholesterol pathways. Similarly, we found that *Lm*-infected Mo-DC exported cholesterol at higher rate than uninfected cells, confirming that *Lm* infection generates a metabolic change in the infected cells that results in an increase of synthesis and export of cholesterol that has not been previously described (Fig. 5B,C).

**Figure 6:**
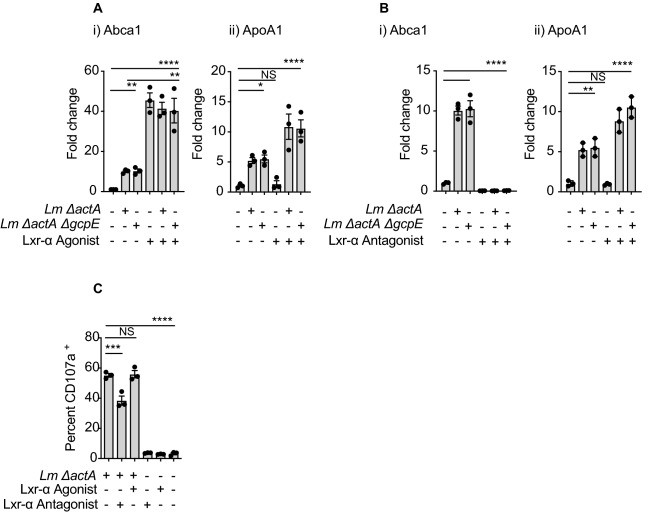
Effects of Lxr-α agonist and antagonist in both *ABCA1* and *APOA1* expression during *Lm* infection and Vγ9Vδ2 T cell activation. Untreated and Mo-DC treated with (**A**) T0901317 -Lxr-α agonist- or (**B**) GSK 2033 -Lxr-α antagonist- were infected with the indicated *Lm* strain or left uninfected. At 6 hpi cells were harvested, RNA extracted and quantitative real time PCR conducted as described in “Methods” section. (**i**) *ABCA1* and (**ii**) *APOA1* gene expression was analyzed and fold changes were expressed as 2^-ΔΔCt^, where the internal control is the *GAPDH* gene and the control samples are uninfected cells. Results shown are representative of at least 3 biologically independent experiments. (**C**) Untreated or pretreated Mo-DC were infected with the indicated *Lm* strain at a MOI = 10 or left uninfected, cells were washed and ex-vivo expanded Vγ9Vδ2 T cells added. Cells were incubated for 18 h, processed and intracellular cytokine staining performed as described in “Methods” section. Left panel, quantitation of percent IFNγ^+^ δ2^+^ CD3 and right panel, flow-cytometry plots are representative results of 4 independent experiments. Data represents the mean ± SEM of each group (n = 3). Statistics calculated by one-way ANOVA with Tukey’s correction; * = *p* < 0.05, ** = *p* < 0.01, **** = *p* < 0.0001, ns = no significant differences observed between the groups analyzed.

Lxr-α is a regulator of lipid homeostasis and inflammation that acts with Lxr-β and/or the retinoid X receptors (RXRs) to control a range of cellular processes^50^. Lxr-α is a sensor for elevated intracellular IPP, and its activation results in increased expression of ABCA1 and ApoA1^49^. We determined that the expression of the *LXR-α* gene is not affected by *Lm* infection in Mo-DC (Suppl Fig. 5D). To address the role of this transcriptional regulator in the expression of *ABCA1* and *APOA1* during *Lm* infection, Mo-DC cells were pre-incubated with T0901317, a synthetic Lxr-α agonist. We similarly found that the *ABCA1* gene was highly upregulated in the presence of T0901317, but we did not find that the expression of *APOA1* was affected by T0901317 (Fig. 6A). Since *Lm* infection was able to induce both genes, this suggests that *Lm* regulates *APOA1* gene expression independent of Lxr-α activation. To confirm these data, we evaluated the expression of these two genes following infection in the presence of the synthetic Lxr-α antagonist GSK 2033. *Lm* infection in Mo-DC could not induce *ABCA1* in the presence of GSK 2033 and its expression was decreased at baseline following Lxr-α inhibition (Fig. 6Bi). GSK 2033 did not affect the expression of *APOA1* and *Lm* infection induced *APOA1* even in the presence of the antagonist (Fig. 6Bii). These data are consistent with *ABCA1* induction during *Lm* infection in Mo-DC being dependent on Lxr-α, but that *APOA1* is regulated through a distinct mechanism. To determine whether regulation of Lxr-α altered Vγ9Vδ2 T cell activation, we treated Mo-DC with the Lxr-α synthetic agonist or antagonist before *Lm*-infection, then evaluated their ability to activate Vγ9Vδ2 T cells. In the absence of *Lm* infection, Lxr-α regulation did not result in Vγ9Vδ2 T cell activation, and the Lxr-α antagonist GSK 2033 only moderately reduced Vγ9Vδ2 T cell activation (Fig. 6C). These data demonstrate that *Lm* access to the cytosol of Mo-DC results in a metabolic shift towards the synthesis and export of cholesterol (and its intermediates) in part via regulation of LXR-α and in part through independent mechanisms. However, manipulation of LXR-α has limited impact on Vγ9Vδ2 T cell activation following infection.

### Lm infection and cholesterol regulatory mechanisms

**Figure 7:**
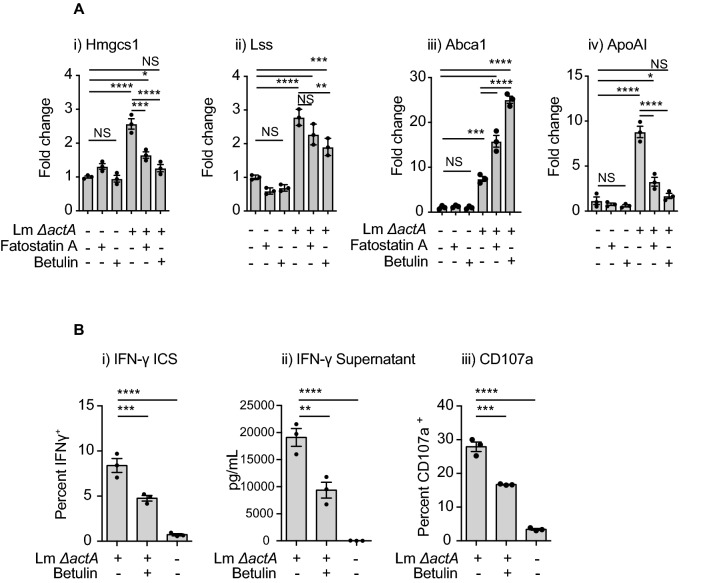
Role of SREBP2 in the gene expression changes observed in *Lm*-infected Mo-DC and Vγ9Vδ2 T cells activation. (**A**) Untreated and fatostatin A or betulin treated Mo-DC were infected with the indicated *Lm* strain or left uninfected. At 6 hpi cells were harvested, RNA extracted and quantitative real time PCR was performed. **i**) *HMGCS1*, **ii**) *LSS*, **iii**) *ABCA1* and **iv**) *APOA1* gene expression was analyzed and fold changes expressed as 2^-ΔΔCt^, where the internal control is the *Gapdh* gene and the control samples are uninfected cells. Results shown are representative of at least 3 biologically independent experiments. (**B**) Untreated and pretreated Mo-DC were infected with the indicated *Lm* strain at a MOI = 10 or left uninfected, cells were washed and ex-vivo expanded Vγ9Vδ2 T cells added. (**i**) Quantitation of percent IFNγ^+^ δ2^+^ CD3. Cells were incubated for 18 h, processed and intracellular cytokine staining performed as described in “Methods” section. (**ii**) Quantitation of IFNγ in the corresponding supernatants by Luminex. (**iii**) Quantitation of percent CD107a^+^ δ2^+^ CD3. Cells were incubated with Vγ9Vδ2 T cells in the presence of an anti-CD107a antibody and monensin. Results shown are representative of at least 3 biologically independent experiments. Data represents the mean ± SEM of each group (n = 3–6). Statistics calculated by one-way ANOVA with Tukey’s correction; * = *p* < 0.05, ** = *p* < 0.01, *** = *p* < 0.001, **** = *p* < 0.0001, ns = no significant differences observed between the groups analyzed.

In view of the increase in transcription of several genes involved in mevalonate/cholesterol biosynthesis and cholesterol export in Mo-DC during the first hours of *Lm* infection we aimed to understand their regulation. Sterol regulatory element-binding protein 2 (SREBP2) is a transcription factor that is primarily responsible for the activation of genes involved in cholesterol synthesis. These include genes that are induced during *Lm* infection, such as *HMGCS1*, *LSS* and *DHCR24*^51,52^. For this reason, SREBP2 is a potential regulator of *Lm* cholesterol pathway gene induction and therefore may regulate Vγ9Vδ2 T cell activation. As expected we found that the expression of *LSS* and *HMGCS1* is increased by *Lm* infection (Fig. 7Ai,Aii), and that the use of two SREBP2 specific synthetic inhibitors, betulin and fatostatin A^53,54^, reduced the upregulation of these genes. These data suggest that SREBP2 activation is involved in sensing *Lm* infection. Both betulin and fatostatin A increase the expression of the *ABCA1* exporter in *Lm* infected Mo-DC (Fig. 7Aiii), suggesting that SREBP2 represses *ABCA1* expression during *Lm* infection. These data agree with those of Zeng et al.,^55^ showing that *ABCA1* expression is downregulated by the SREBP2 transcriptional regulator in endothelial cells^55^. Conversely, betulin or fatostatin A blocks *APOA1* upregulation during *Lm* infection of Mo-DC, suggesting that SREBP2 mediates Lxr-α-independent regulation of *APOA1* following *Lm* infection (Fig. 7Aiv). Importantly, SREBP2 inhibition during infection reduced Vγ9Vδ2 T cell activation (Fig. 7Bi,Bii,Biii). Thus, SREBP2 is an important sensor detecting *Lm* infection of Mo-DC that regulates Vγ9Vδ2 T cell activation by infected cells. It was recently described that betulin could inhibit *Lm* invasion of bone marrow derived macrophages and LDH release after *Lm* infection^56^. However, we have not found any change in ability of *Lm* to infect Mo-DC cells in the concentrations and time points evaluated (Suppl Fig. 5C).

**Figure 8:**
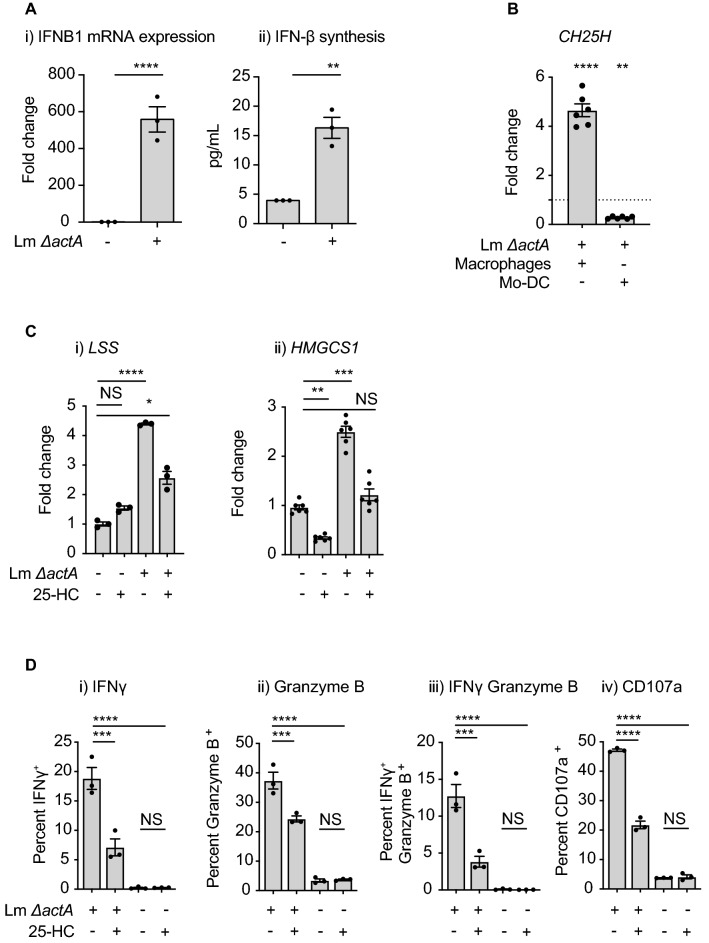
Role of 25-HC in the gene expression changes observed in *Lm*-infected Mo-DC and Vγ9Vδ2 T cell activation. (**A**) Mo-DC were infected with *Lm* or left uninfected and at 6 hpi cells were harvested for RNA extraction and supernatants kept for IFN-β quantification. **i**) qRT-PCR analysis of the expression of the *IFNB1* gene and **ii**) IFN-β quantification. (**B**) Human myeloid derived macrophages and Mo-DC were infected with *Lm* at MOI = 10, at 6 hpi cells were harvested for RNA extraction and qRT-PCR analysis of the expression of the *CH25H* gene was done. Fold changes are expressed as 2^-ΔΔCt^, where the internal control is the *Gapdh* gene and the control samples are uninfected cells. (**C**) Untreated and 25-HC (10 μM) treated Mo-DC were infected with the indicated *Lm* strain (MOI = 10) or left uninfected. At 6 hpi cells were harvested, RNA extracted and quantitative real time PCR conducted as described in “Methods” section. (**i**) *LSS* and (**ii**) *HMGCS1* gene expression was analyzed and fold changes expressed as 2^-ΔΔCt^, where the internal control is the *Gapdh* gene and the control samples are uninfected cells. Results shown are representative of 3 biologically independent experiments. (**D**) Untreated and 25-HC pretreated Mo-DC were infected with the indicated *Lm* strain at a MOI = 10 or left uninfected, cells were washed and ex-vivo expanded Vγ9Vδ2 T cells added. Cells were incubated for 18 h, processed and intracellular cytokine staining performed as described in “Methods” section for quantification of percent (**i**) IFNγ^+^ δ2^+^ CD3^+^, (**ii**) Granzyme B^+^ δ2^+^ CD3^+^ and (**iii**) IFNγ^+^ Granzyme B^+^ δ2^+^ CD3^+^. (**iv**) Mo-DC were incubated with Vγ9Vδ2 T cells in the presence of an anti-CD107a antibody and monensin and CD107a^+^ δ2^+^ CD3^+^ quantified. Results shown are representative of at least 3 biologically independent experiments. Data represents the mean ± SEM of each group (n = 3). Statistics calculated by unpaired *t* test and one-way ANOVA with Tukey’s correction; ** = *p* < 0.01, *** = *p* < 0.001, **** = *p* < 0.0001, ns = no significant differences observed between the groups analyzed.

Entry of *Lm* into the cytosol also triggers innate sensors resulting in type I IFN transcription^57–60^. Devilder et al.,^61^ demonstrated that TLR3 or TLR4-activated Mo-DC were able to trigger IFN-γ production by Vγ9Vδ2 T cells through the Type I IFN production. To exclude IFN-β-related effects, we used a blocking antibody and demonstrated that IFNAR2 blockade had no effect on Vγ9Vδ2 T cell activation (Suppl Fig. 5B). Type I IFN signaling can regulate cholesterol synthesis via regulation of *CH25H* which codes for 25-hydroxycholesterol oxidase, an enzyme that catalyzes the synthesis of the oxysterol 25-Hydroxycholesterol (25-HC)^52,62^. 25-HC is a metabolic inhibitor of SREBP2 activity^63^ and is an endogenous ligand for Lxr-α^64^. In Mo-DC we observed increased transcription of *ifnβ* following *Lm*-infection, as well as release of IFN-β in the supernatants (Fig. 8Ai,ii). Interestingly, infection of Mo-DC downregulated *CH25H* expression (Fig. 8B), suggesting that Type I IFN are not coordinately regulated *CH25H* as has been reported in other cell types. We performed a similar experiment in human macrophages and found increased expression of *CH25H* following infection (Fig. 8B), in agreement with the published studies using *Lm*-infected murine macrophages^65,66^. These data suggest that human macrophages and human dendritic cells differently regulate *CH25H* following infection. To test the consequence of *CH25H* gene activity in Mo-DC we treated cells with 25-HC during infection. 25-HC treatment during *Lm* infection of Mo-DC reduced the expression of the *HMGCS1* and *LSS* genes and Vγ9Vδ2 T cell activation (Fig. 8C,D). These results are consistent with this pathway acting as a negative regulator of Vγ9Vδ2 T cell activation but that it is not functional in Mo-DC during the initial steps of *Lm* infection. Together, these data implicate SREBP2 as a critical regulator activated following *Lm* infection that is necessary for metabolic changes in the infected cell and activation of Vγ9Vδ2 T cells that can occur in the absence of HMBPP production in the bacteria.

## Discussion

In this study we show that in human dendritic cells, the entry of *Lm* into the cytosol drives a change in gene expression and a metabolic shift towards cholesterol synthesis and export. These changes have not been previously reported and result in increased recognition of infected cells by a pre-existing population of Vγ9Vδ2 T cells. This in turn results in dendritic cell death as well as locoregional cytokine production. Due to this activation of endogenous cholesterol metabolism in the infected cells, recognition by Vγ9Vδ2 T cells does not require HMBPP production from the infecting bacterium. Kistowska et al., previously demonstrated that human monocytes and immature DC infected with *E. coli* or *Staphylococcus aureus*, bacterium that synthesize or lack HMBPP respectively, were both able to activate Vγ9Vδ2 T cells^16^. In agreement with our observations, Vγ9Vδ2 T cell activation in the early stages of infection involving changes in the first steps of the mevalonate pathway through increased cellular protein levels and activity of the HMGCR enzyme that would lead to increasing levels of intracellular IPP. While we did not detect changes in transcription of the *HMGCR* gene, we did detect changes in the transcription of several genes involved in the mevalonate/cholesterol biosynthesis and export.

In our model, where *Lm*-infected Mo-DC are put in close contact with Vγ9Vδ2 T cells and their activation and cytotoxicity were analyzed very early following infection, we demonstrate that the HMBPP metabolite is unnecessary for the activation of Vγ9Vδ2 T cells during an active infection. However, later activation of Vγ9Vδ2 T cells while still independent of HMBPP is only partially affected by BTN3A1 blockade. These data suggest that additional factors resulting from infection contribute to Vγ9Vδ2 T cell activation. *Brucella*-infected Mo-DC have been shown to induce a similarly late IFNγ production in Vγ9Vδ2 T cells, which in turn results in full maturation of the infected Mo-DC and IL-12 production^43^, and a similar mechanism has been described following S. aureus infection^67^. IL-12 released from *S. aureus*-infected Mo-DC contributes to rapid IFNγ production by Vγ9Vδ2 T cells^67^ This effect requires close contact between the infected cells and Vγ9Vδ2 T cells and is γδTCR dependent, confirming that in this bacterium that does not produce HMBPP endogenous IPP could explain Vγ9Vδ2 T cell activation. IL-12 has been shown to activate Vγ9Vδ2 T cells in combination with TCR ligation^42^, and independently of TCR ligation as a recombinant cytokine^68^, and may explain late HMBPP-independent Vγ9Vδ2 T cell activation. In our experiments NKG2D, a well-known co-stimulator of Vγ9Vδ2 T cells, does not appear to contribute to the observed activation or cytotoxicity. These data agree with some related studies, for example *Mycobacterium tuberculosis*-infected Mo-DC were able to trigger Vγ9Vδ2 T cell proliferation and perforin synthesis that was not affected by an anti-NKG2D blocking antibody^69,70^. However, other studies demonstrate that Vγ9Vδ2 T cell-mediated lysis of *M. tuberculosis*-infected Mo-DC and Brucella-infected DC is partially affected by NKG2D blockade^44,45^. These conflicting data suggest that there may be specific model-dependent features that dictate whether NKG2D contributes to Vγ9Vδ2 T cell function.

Prior studies with *Lm* demonstrating the relevance of HMBPP used in vitro models where either bacterial extracts or supernatants of infected neutrophils were applied to PBMCs or monocytes^34,36–38^. These two types of experimental designs do not model the metabolic changes that occur in the host cell that could affect Vγ9Vδ2 T cell activation. In non-human primates HMBPP negative strains are less able to expand Vγ9Vδ2 T cells when infected via an intranasal route, but Vγ9Vδ2 T cell expansion still occurs^40,41^. It is interesting to speculate whether the route of infection and therefore the cells that are infected impacts the lytic release of metabolites and the dependence on HMBPP production by the bacterium. In mice, oral infection can generate a distinct pattern of immune activation to IV infection with the bacterium^71–73^.

Our studies suggest that the cell type that is studied dramatically impacts the metabolic response to *Listeria* infection. Previous studies in *Lm*-infected murine bone marrow-derived macrophages have shown a reduced expression of the *ABCA1* gene as well as other Lxr-α responsive genes^74^. Our finding of the concerted induction of the *ABCA1* gene together with that of the *APOA1* gene in Mo-DC provides evidence for the differential regulation in the different cell types analyzed. Furthermore, in murine bone marrow-derived macrophages and macrophage cell lines, *CH25H* is induced following *Lm* infection as a consequence of increased synthesis of Type I IFN^65,66,75^. In human Mo-DC we showed no induction of *CH25H* gene expression during the first hours of *Lm* infection even though *ifnβ expression* is induced and IFN-β is synthesized and secreted. 25-HC, the product of the *CH25H* gene acts as a repressor of genes involved in sterol biosynthesis via a reduction of the active form of the SREBP2 regulator and the consequent decreased accumulation of cholesterol^65^. Since *Lm*-infected human Mo-DC do not induce *CH25H*, our results match *CH25H* knockout macrophages^65,66^, where the absence of induction of *CH25H* expression leads to the upregulation of several genes involved in the mevalonate/cholesterol pathway that are under the control of the SREBP2 regulator. Consequently, in DC cholesterol accumulation leads to activation of the LXR-RXR that results in the observed *ABCA1* upregulation and cholesterol export. It is our hypothesis that the intracellular levels of the pAg IPP are increased during this metabolic shift. Consistent with Castella et al*.,*^49^ this metabolite could either act as activator of the Lxr-α transcriptional regulator and/or that IPP is being exported to the microenvironment through the ABCA1-ApoA1 complex. Alternatively, since we have confirmed that *BTN3A1* expression in Mo-DC is essential for full activation of Vγ9Vδ2 T cells by *Lm*, it is conceivable that the intracellular IPP/DMAPP could directly bind the intracellular domain of this protein leading to the direct Vγ9Vδ2 T cell activation by *Lm*-infected Mo-DC. Notably, we determined that the expression of the *APOA1* gene is also greatly increased during *Lm* infection. In contrast to results described previously using relatively undifferentiatied myeloid populations^49^, in our system using fully differentiated Mo-DC the *APOA1* gene appears not to be under the control of the Lxr-α regulator. Our results obtained using SREBP2 inhibitors betulin and fatostatin A strongly suggest that SREBP2 upregulates the transcription of the *APOA1* gene during *Lm* infection.

Remarkably, the observed changes in expression of the genes involved in cholesterol synthesis and export, as well as the consequent Vγ9Vδ2 T cells activation, depend on the bacterial escape from the phagolysosome and cytoplasmic replication. Lysteriolysin O (LLO), a cholesterol-dependent cytolysin encoded by the *hly* gene, allows bacterial escape from the phagolysosome to the cytosol where it can replicate without causing appreciable antigen presenting cell death^27^. Monk et al.^76^, previously showed that human monocytes infected with a *hly* deficient *Lm* strain were able to stimulate Vγ9Vδ2 T cell proliferation similarly to that of the wild type strain. Given the significant differences in bacterial lysis by macrophages versus dendritic cells, it is possible that cytoplasmic entry is not a critical component to generate a Vγ9Vδ2 T cell response where the bacteria are rapidly cleared.

Statins inhibit 3-hydroxy-3-methylglutaryl-coenzyme A reductase (HMGCR), the rate-limiting enzyme in the synthesis of mevalonate^77^, which is a necessary precursor for synthesis of IPP, cholesterol and isoprenoids. Bisphosphonates such as zoledronic acid and risedronate are able to increase the intracellular IPP pool by inhibiting the farnesyl pyrophosphate synthase (FPPS) in the mevalonate pathway^78,79^. It has previously been shown that antigen presenting cells that are preincubated with the bisphosphonate zoledronic acid activated Vγ9Vδ2 T cells due to increased intracellular IPP^78,80–82^. By contrast, antigen presenting cells preincubated with the statin mevastatin limited Vγ9Vδ2 T cell activation due to decreased IPP. Importantly, HMBPP activation of Vγ9Vδ2 T cells has been shown to be resistant to statins^14^, and critically, the *Lm* HMGCR is weakly inhibited by the statins, requiring 1000-fold higher concentrations to observe effects^83^. These data strongly suggest that our results demonstrating decreased Vγ9Vδ2 T cell activation as a result of limiting the endogenous mevalonate pathway either by the use of statins or SREBP2 inhibitors during *Lm* infection, support HMBPP-independent function. While mevastatin was able to completely inhibit the CD107a expression in Vγ9Vδ2 T cells that occurred following risedronate or zoledronic acid treatment, mevastatin only partially reduced CD107a expression in Vγ9Vδ2 T cells incubated with *Lm*-infected Mo-DC. This may be due to *Lm* infection also inducing the expression of other genes involved in the mevalonate/cholesterol pathway that are not inhibited by mevastatin, which are not induced by bisphosphonates. Alternatively, bacterial IPP/DMAPP could contribute to this observed statin-independent activation. Further study of the host metabolic response to infection is necessary to characterize this hypothesis. Although we cannot rule out the possibility that other factors could be involved, our data suggest that the mevastatin-mediated reduction in Vγ9Vδ2 T cell activation is primarily a consequence of the expected changes in the intracellular IPP pool or that of its isomer DMAPP. These effects of blocking endogenous mevalonate pathway occur despite the presence of bacterial HMBPP synthesis through the alternative mevalonate pathway, indicating that even in bacteria where this pathway is fully functional, the endogenous mevalonate pathway is the dominant mechanism for Vγ9Vδ2 T cell activation in infected dendritic cells.

Together, these data place host cell metabolic regulation at the center of the immune response to *Lm* infection, and given the central importance of Vγ9Vδ2 T cell responses in infected humans, the impact of pathway regulators such as statins becomes highly relevant. However, as discussed earlier gcpE-deleted *Lm* have shown decreased ability to activate Vγ9Vδ2 T cell responses in primate studies^39–41^, suggesting that HMBPP production is playing a role in the in vivo response to *Lm* vaccination. Given the complicated interactions that occur between the more abundant lysteriolytic cells such as neutrophils and macrophages, and the critically important role of direct infection by less abundant dendritic cells for lymphocyte activation, the interplay between indirect lytic release and direct infection may be highly impactful in the response to in vivo bacterial infection.

## Methods

### Bacterial strains and construction

*Lm* strains used for these studies are all derived from the attenuated *ΔactA* strain DP-L4029^84^. When indicated we used the *Δhly* DP-L4027 strain^84^. Strains were grown in brain–heart infusion broth, washed and diluted in PBS before infecting Mo-DC or macrophages at the indicated MOI. All nonpolar gene deletions were generated by allelic exchange using the temperature-sensitive plasmid pKSV7 as described^85,86^.

Sequences located up- and downstream of the corresponding genes were amplified with Q5 High Fidelity DNA polymerase (NEB, Ipswich, MA) using primers whose sequences are described in Supplementary Table 1. Amplified fragments were cloned in the pKSV7 vector and were introduced into TOP10 *E. coli* (Invitrogen, Carlsbad, CA). Identified constructs were confirmed by sequencing, plasmids were then transformed into SM10 *E. coli* and conjugated into *Lm* strains. Positive clones were identified by performing colony PCR with Q5 High Fidelity DNA polymerase (NEB, Ipswich, MA).

### Generation of monocyte-derived dendritic cells (Mo-DCs)

To generate monocyte-derived DCs, human peripheral blood mononuclear cells (PBMC), obtained from healthy donors, were isolated by Ficoll^®^ Paque gradient centrifugation (GE Healthcare Bio-Sciences Corp., Piscataway, NJ) from buffy coats. The protocol for collection of de-identified PBMC from health donors was approved by the Providence Health and Services Institutional Review Board (IRB#06-108), was carried out in accordance with relevant guidelines and regulations, and all participants provided written informed consent. CD14 + monocytes were positively selected using EasySep™ Human CD14 positive selection (StemCell,Vancouver BC,Canada, cat # 17858). Purified monocytes were cultured at a density of 10^6^ cells/ml in 6-well plates for 5 days in RPMI-HS [RPMI 1640 (GE Healthcare, cat# SH30027.LS) containing 5% heat inactivated human serum (HS, Valley Biomedical, Winchester, VA, cat # HS1021 HI), 2.05 mM L-glutamine] with the addition of 50 ng/ml recombinant human granulocyte–macrophage colony stimulating factor (GM-CSF, Leukine^®^ Sargramostim, Sanofi-Aventis, Bridgewater, NJ,) and 20 ng/ml recombinant human interleukin 4 (IL-4, R&D Systems, Minneapolis, MN, cat # 204-IL-050/CF) to generate Mo-DC. Cytokines were replenished on day 3 and cells harvested on day 5.

### Determination of viable counts

Mo-DCs were collected prior to infection and plated at a density of ~ 5 × 10^5^ cells/ml. *Lm* strains were grown in BHI media, OD_600nm_ measured, washed twice with PBS and added to Mo-DC at the desired MOI (usually ~ 10). Cultures were incubated 1 h at 37 °C 5% CO_2_ to allow the bacteria to attach and invade the cells. Wells with cells were washed with PBS, fresh RPMI-HS media containing 50 μg/ml gentamycin was added and incubation continued. Infected Mo-DC were lysed at various time points with 0.2% v/v Igepal (Sigma-Aldrich, Saint Louis, MO), diluted in PBS, plated on BHI plates and incubated overnight at 37 °C to determine the colony forming units (CFU).

### Vγ9Vδ2 T cell ex-vivo expansion

PBMC from healthy adult donors were obtained and processed as described above. For ex vivo expansion experiments, complete media (C-Media) was used and prepared with OpTmizer (Invitrogen, Carlsbad, CA), supplemented with 10 mM HEPES, 1 mM L-glutamine, 10% human serum (Valley Biomedical, Winchester, VA, cat # HS1021 HI). For expansions performed in 24-well plates, 1 × 10^6^ PBMCs were suspended in 1 ml of media and incubated with 5-10 μM zoledronate (ZA) (Zometa® zoledronic acid, Novartis, Basel, Switzerland). After 4 h the plates were centrifuged and the media with zoledronate removed. The cells were washed and then 1 ml of fresh C-media was added. On day 3, 500 μl of media was removed and replaced with media containing 1000U IL-2 (PeproTech Inc., Rocky Hill, NJ). PBMC were incubated for 14 days at 37 °C and 5% CO_2_. Cell growth was monitored by microscope examination and media color. Every 2 to 4 days, 50% of the media was changed for fresh media (containing IL-2 but without zoledronate) and the cells were split 1:2 depending on cell density. On day 14, the cells were harvested, washed and counted. Levels of γδ and Vγ9Vδ2 T cells and their differentiation state (CD27 and CD45RA) were assessed by flow cytometric analysis. Further purification (> 90%) of expanded Vγ9Vδ2T cells or naïve Vγ9Vδ2 T cells was performed with TCRγ/δ^+^ T Cell Isolation Kit (Miltenyi Biotec, Bergisch Gladbach, Germany). Repeated analysis of ex vivo expanded Vγ9Vδ2 T cells demonstrated greater than 90% viability of purified cells and minimal expression of the activation markers CD25, IFNg, CD107a, and Granzyme B without further stimulation. Further analysis of Vγ9Vδ2T cell activation was limited to gated populations and minimal activation of contaminated populations was detectable.

### Vγ9Vδ2 T cell activation

Human Mo-DCs obtained as described above were plated in RPMI-HS in round bottom 96-wells plates (~ 4–6 × 10^4^ cells), infected with the indicated *Lm* strain at a MOI = 10, or the indicated MOI, and plates centrifuged 4 min at 400 g. After 1 h of incubation at 37 °C, cells were washed twice to remove extracellular bacteria and 100 μl of fresh media including gentamycin 50 μg/ml was added. Purified γ9δ2 T cells that were either ZA-expanded or naïve were added to each well and incubated at 37 °C and 5% CO_2_. Ratio Mo-DC: Vγ9Vδ2 T cells was usually 1:1, otherwise the ratio used is indicated in the text or figure. Unless indicated in the text, media was removed after 18–20 h of incubation, new fresh media was added including brefeldin A and after 4 h cells were washed and stained as described in “Flow cytometry” section.

When different compounds were tested in the activation of the γ9δ2 T cells, Mo-DCs were incubated overnight with the indicated compound, cells were infected with *Lm* or left uninfected, washed and Vγ9Vδ2 T cells added as described above. For NKG2D blocking assays, Vγ9Vδ2 T cells were pre-incubated with anti-NKG2D antibody (clone 1D11, Millipore Sigma, Burlington, MA). Similarly for IFNAR2 blocking of the Vγ9Vδ2 T cells activation, these cells were incubated with various concentrations of the anti-IFNAR2 antibody (clone MMHAR-2, Millipore Sigma, Burlington, MA).

For BTN3A1 blocking, human Mo-DCs obtained as described were plated in RPMI-HS in round bottom 96-wells plates (~ 4–6 × 10^4^ cells), infected with the indicated *Lm* strain at a MOI = 10 and plates centrifuged 4 min at 400 g. After 1 h of incubation at 37 °C, cells were washed twice to remove extracellular bacteria and 100 μl of fresh media including 50 μg/ml gentamycin and either 10 μg/ml anti-BTN3A1 blocking antibody^19^ (clone 103.2, Creative Biolabs, Shirley, NY) or 10 μg/ml IgG isotype were added. After 45 min of further incubation at 37C, purified Vγ9Vδ2 T cells were added to each well and incubated at 37 °C and 5% CO_2_. Ratio Mo-DC: Vγ9Vδ2 T cells was 1:1. Cells were incubated another 2 h in presence of monensin for CD107a staining. For IFNγ and CD25 staining media was removed after 18–20 h of incubation, new fresh media was added including brefeldin A and after 4 h cells were washed and stained as described in below.

When bacterial extracts were evaluated, *Lm* strains were grown in liquid BHI, harvested at an optical density (OD)_600 nm_ of 0.8, and sonicated in 1/10 volume phosphate-buffered saline, pH7.4. Low molecular weight (LMW) fractions were obtained using Centriprep 3 kDa filters (Millipore Sigma, Burlington, MA). PBMCs were plated at approximately 1 × 10^5^/well and LMW samples were tested at serial dilutions between 1:2 and 1:10^5^. Cells were harvest after 72 h, stained and analyzed for the expression of CD25.

### Flow cytometry

For staining, cells were stained with Zombie Aqua Viability Dye (#423102, BioLegend, San Diego, CA) in PBS for 10 min on ice, then Fc receptors were blocked with Human BD Fc Block™ from BD Biosciences (#564219, BD Biosciences, San Jose, CA) for an additional 10 min. After centrifugation, the supernatant was removed and cells were stained with a surface antibody cocktail containing in FACS buffer (PBS, 2 mM EDTA, 2% FBS) and, if needed, Brilliant Stain Buffer Plus from BD Biosciences (#566385) for 20 min on ice. After surface staining, cells were washed in FACS buffer and fixed for 20 min on ice with Fixation/Permeabilization Buffer from BD Biosciences (#554722). For intracellular cytokine analysis, single cell suspensions were incubated in RPMI + 5% HS and 10 ug/mL GolgiPlug from BD Biosciences (#555029) at 37 °C for 4 h. Cells were then stained as described above and afterwards cells were incubated with intracellular antibodies for 30 min on ice. All samples were resuspended in FACS buffer and acquired on a BD Fortessa or a BD LSRII flow cytometer. Data was analyzed using FlowJo software from Tree Star, v10.5.

The following antibodies were purchased from BioLegend (San Diego, CA); CD25-BV711 (M-A251), CD107a-PE (H4A3) and TNF-α BV605 (Mab11). CD11c-PE/Cy7 (B-ly6), IFN-γ BV650 (B27), CD25-APC (M-A251), CD69 APC Cy7 (FN50), Granzyme-B BV421 (GB11), CD14-AF700 (M5E2), Perforin PE-CF594 (δG9), Vγ9-PE (B3), CD38-PE (HIT2), CD40-PE (5C3), CD86-APC (2331), CD40L-PE/Dazzle™ 594 (24–31) and HLA-DR-V450 (G46-6) were obtained from BD Biosciences (San Jose, CA). TCR Vδ2-FITC (123R3) was obtained from Miltenyi Biotec (Bergisch Gladbach, Germany) and CD3-AF700 (UCHT1) was obtained from Thermo Fisher Scientific, Waltham, MA.

### Reagents

25-Hyroxycholesterol (#5741), T0901317 (#2373), GSK 2033 (#5694) and fatostatin A (#4444) were obtained from Tocris Bioscience (Minneapolis, MN), botulin (#T312) from TargetMol (Wellesley Hills, MA), mevastatin from Sigma-Aldrich (St. Louis, MO). All reagents were suspended in the recommended solvent, aliquoted, kept at -20C and used at the concentrations described in the text or the figures.

### RNA extractions and qRT-PCR

For RNA extraction and quantitative Real time-PCR (qRT-PCR), Mo-DC or human macrophages were plated in a 24-well plate (5 × 10^5^ cells per well) and infected with the indicated *Lm* strains for 1 h, washed and suspended in RPMI-HS media including gentamycin 50 μg/ml. At various time points cells were harvested and RNA was purified using Qiazol (Qiagen, Valencia, CA) and Direct-zol RNA miniprep kit (Zymo Research, Irvine, CA). DNase-treated RNA was used as template for cDNA synthesis using SuperScript™ IV VILO™ Reverse Transcriptase (Invitrogen, Carlsbad, CA) and qRT-PCR was performed using iTaq™ Universal^®^ SYBR Green Supermix (Bio-Rad, Hercules, CA) and primers described in Supplementary Table 1). Reactions were carried out and analyzed in a StepOnePlus™ Real-Time PCR system (Applied Biosystems, Foster City, CA). Fold change was expressed as 2^-ΔΔCt^, where the internal control is the *Gapdh* gene and the control samples are uninfected cells.

### Cholesterol quantification and cholesterol efflux

Cholesterol was quantified in *Lm*-infected or uninfected Mo-DC using the Choelsterol/Cholesteryl ester assay kit as described by the manufacturer (#ab65359 Abcam, Cambridge, MA). In general infected cells were processed after 6 h post-infection. Cholesterol efflux was determined using the Cholesterol efflux assay kit (ab196985, Abcam, Cambridge, MA) following manufacturer’s instructions and the recommended positive control.

### Cytokine luminex assay

Supernatants were collected at the indicated time points, debris removed by centrifugation at 14,000 g for 15 min at 4 °C, and stored in aliquots at -80. IFNγ and IFNβ were detected using 25 μl of supernatants and the ProcartaPlex Human Basic Kit (#EPX010-10,420–901, Invitrogen, Carlsbad, CA) and Procarta Plex Human IFN beta Simplex (#EPX01A-12088-901, Invitrogen, Carlsbad, CA) respectively. Data was acquired on a Luminex 100 array reader and cytokine concentrations for each sample was calculated using standard curves for each analyte.

### Statistics

Data were analyzed and graphed using Prism Version 9.1.0 March 15, 2021 (GraphPad Software, La Jolla, CA). Normal distribution was assessed by using Shapiro–Wilk test. Individual data sets were compared using Student’s *t*-test and analysis across multiple groups was performed using ANOVA with individual groups assessed using Tukey’s comparison.

## Supplementary Information

Supplementary Information.

## Abbreviations

Mo-DC: Monocyte-derived dendritic cells
HMBPP: (E)-4-hydroxy-3-methyl-but-2-enyl pyrophosphate
