# Supplementary material for: Listeria monocytogenes-infected human monocytic derived dendritic cells activate Vγ9Vδ2 T cells independently of HMBPP production
Source: Sci Rep. 2021 Aug 11;11:16347. doi: 10.1038/s41598-021-95908-5 (PMC8358051; doi:10.1038/s41598-021-95908-5)
Supplement: Supplementary file 1 — Supplementary Information. [file 41598_2021_95908_MOESM1_ESM.pdf]

## Supplementary Information for

# *Listeria monocytogenes*-infected human monocytic derived dendritic cells activate Vγ9Vδ2 T cells independently of HMBpp production.

Alejandro F. Alice [1], Gwen Kramer [1], Shelly Bambina [1], Keith S. Bahjat [2], Michael J. Gough [1] and Marka Crittenden [1,3,4]

[1] Earle A. Chiles Research Institute, Robert W. Franz Cancer Center, Providence Portland Medical Center, 4805 NE Glisan St, Portland, OR, 97213, USA.

[2] Current address: Astellas Pharma US, 100 Kimball Way South San Francisco, CA, 94080.

[3] The Oregon Clinic, Portland, OR, 97213, USA

[4] Correspondence to: marka.crittenden@providence.org

a) Mevalonate and MEP pathway

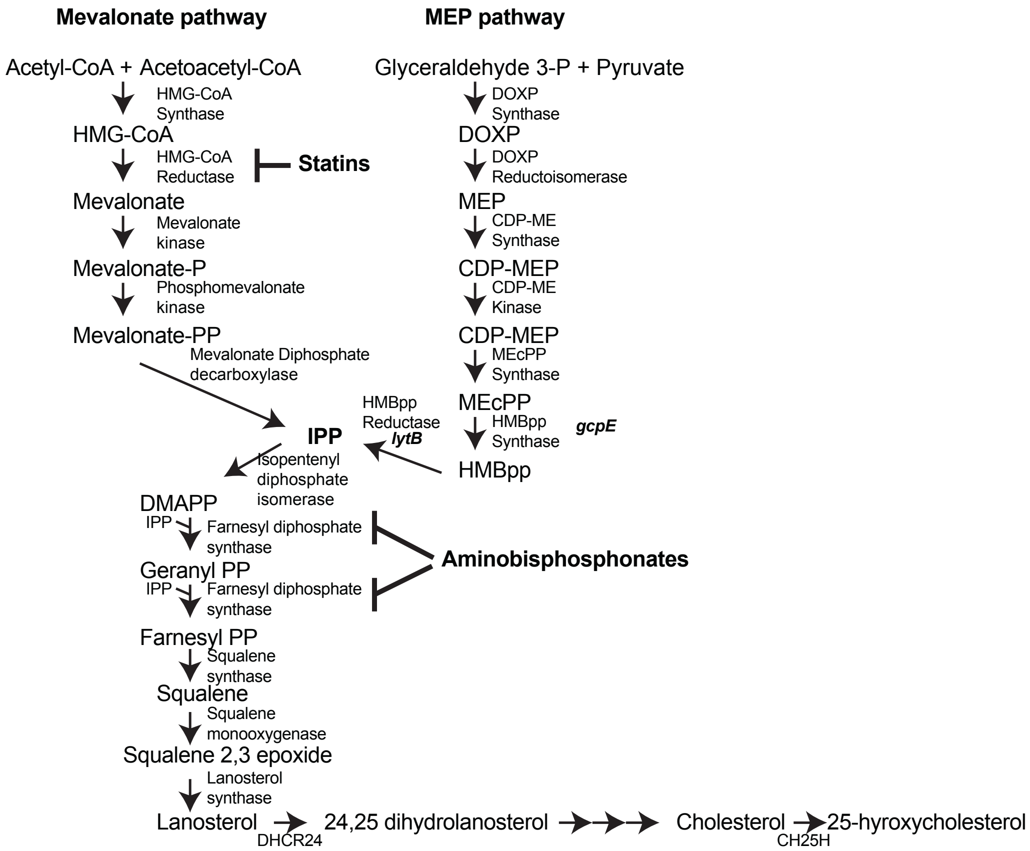


b) Confirmation of the mutations in *Lm* strains

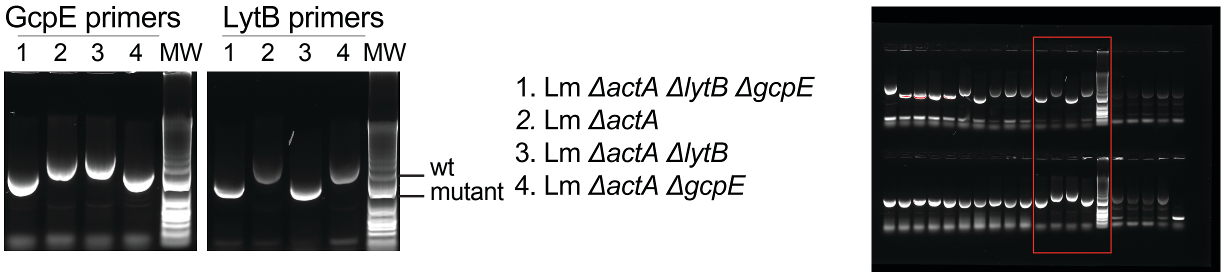


Suppl Figure 1. **A**) Schematic of the mevalonate and the 2-*C*-methyl-D-erythritol 4-phosphate/1-deoxy-D-xylulose 5-phosphate (MEP) or non-mevalonate (only present in *Lm*) pathways. The points of regulation by statins or aminobisphosphonates in the host cells are indicated. **B**) Electrophoresis showing the PCR results obtained using the indicated primers for the *gcpE* and *lytB* genes. Original gel on the right. The genomic DNA from each *Lm* strain used as template is also indicated.

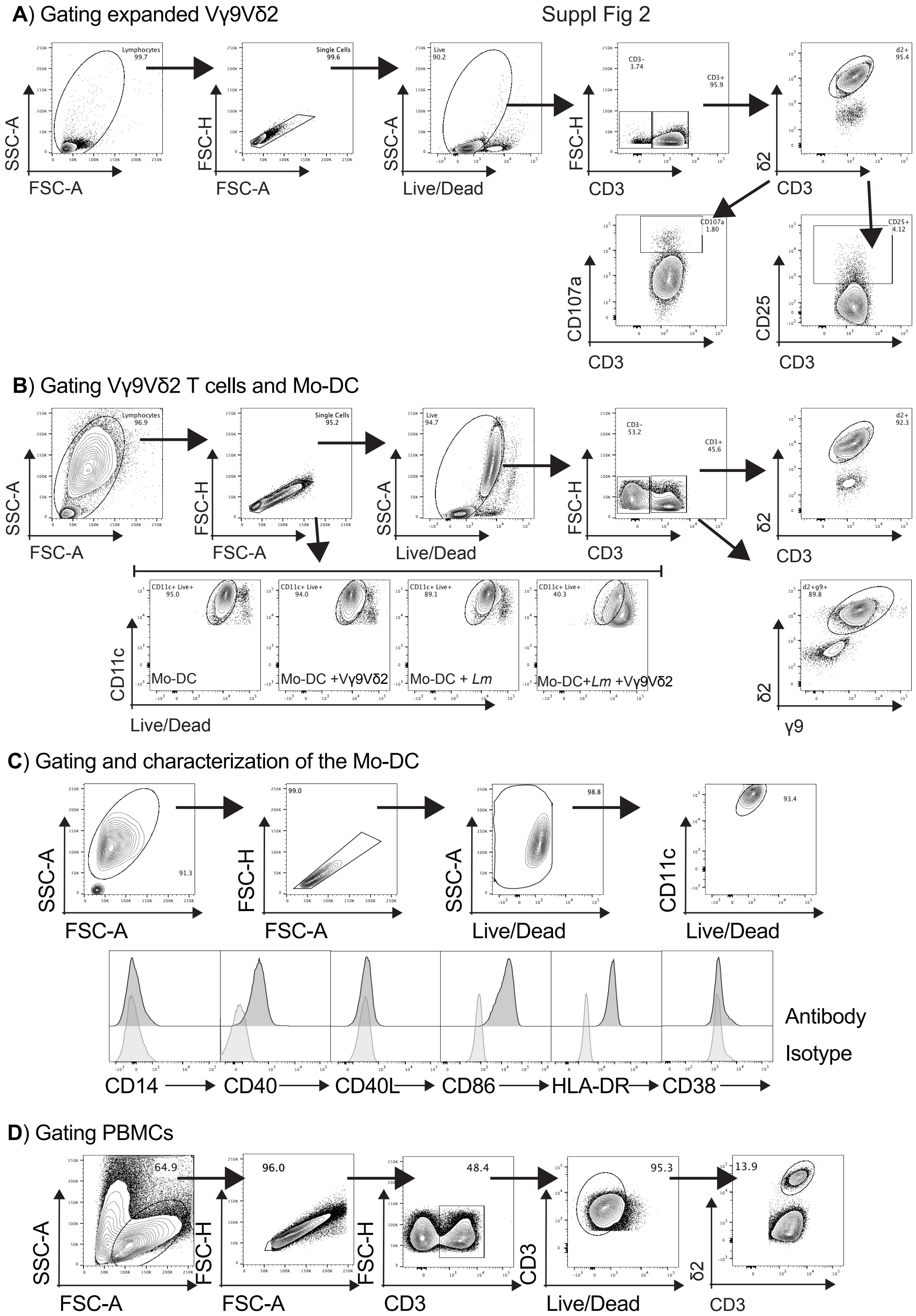


Suppl Figure 2. **A**) Gating strategy for the expanded and purified <u>Vγ9Vδ2</u> T cells. **B**) Gating strategy for Lm-infected Mo-DC incubated with <u>Vγ9Vδ2</u> T cells . **C**) Gating

strategy for the Mo-DC and their phenotypic characterization after 5 days of incubation in the presence of GM-CSF and Il-4 as described in Materials and Methods. **D**) Gating strategy for the PBMCs used in the experiments described in Fig 1. A.

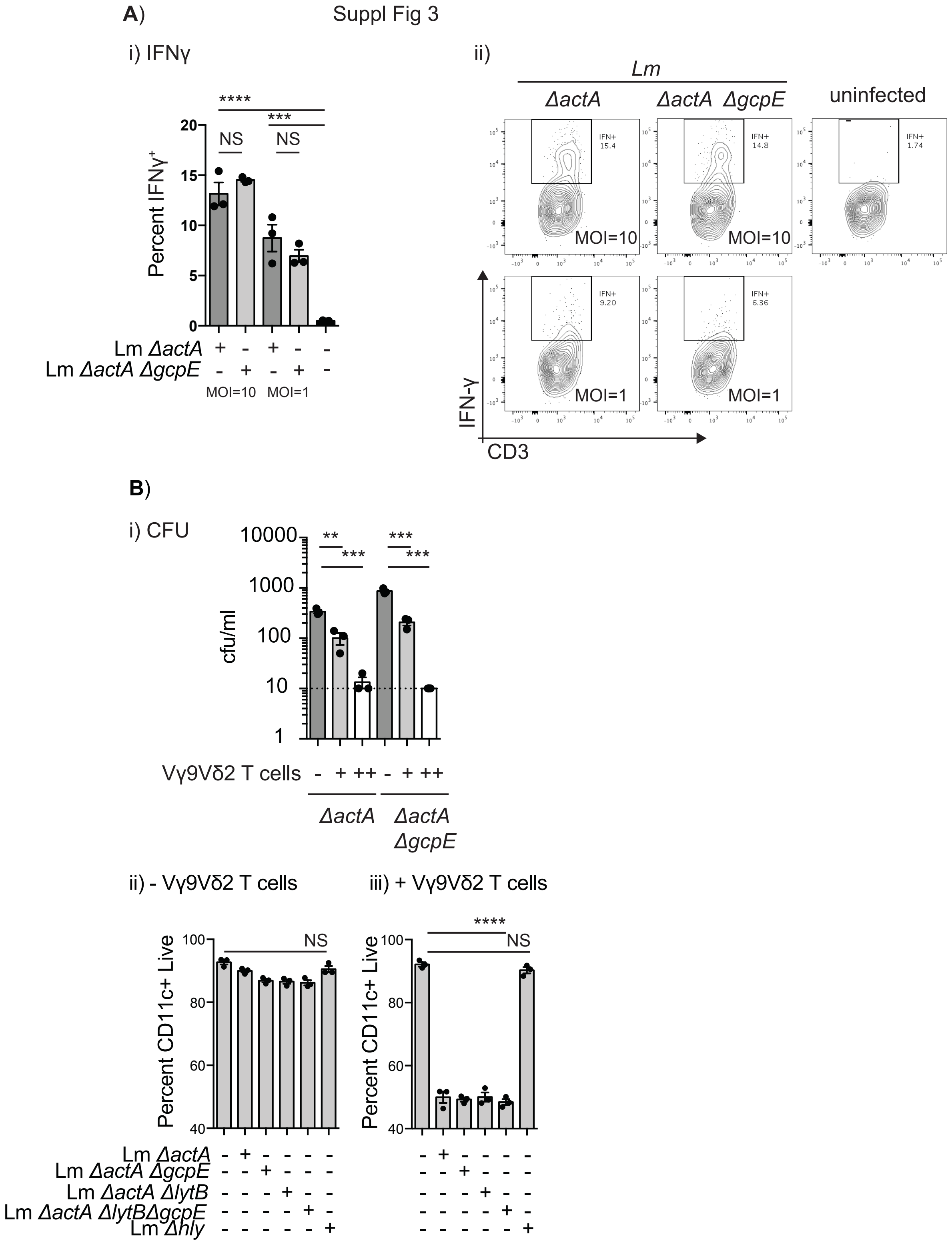


Suppl Figure 3. **A**) Analysis of the IFNγ production by expanded Vγ9Vδ2 T cells using various bacterial MOI. Mo-DC were infected at different MOI with the indicated *Lm* strain

or left uninfected, cells were washed and ex-vivo expanded Vγ9Vδ2 T cells added. Cells were incubated for 18 h, processed and intracellular cytokine staining performed as described in Material and Methods. **Ai**) Quantitation of percent IFN$\gamma^+$ $\delta 2^+$ CD3, **Aii**) Representative flow-cytometry plots. **B**) Analysis of the cytotoxicity against *Lm*-infected Mo-DC. **Bi**) Determination of viable counts when different ratios Mo-DC Vγ9Vδ2 T cells were used. The ratios Mo-DC: Vγ9Vδ2 T cells used in these experiments were 10:1 (+) and 1:1 (++). The symbol -, indicates that Vγ9Vδ2 T cells were not added to the infected cells. The dotted line indicates the limit of detection. **Bii** and **Biii**) Cytotoxicity against Mo-DC infected with various *Lm* strains. **Bii**) Quantitation of the CD11c+ Live+ cells infected with the indicated *Lm* strain or left uninfected in the absence or **Biii**) in the presence of Vγ9Vδ2 T cells. Data represents the mean ± SEM of each group. Statistics calculated by one-way ANOVA with Tukey's correction; ** = $p<0.01$, *** = $p<0.001$, **** = $p<0.0001$, ns = no significant differences observed between the groups analyzed.

Suppl Fig 4

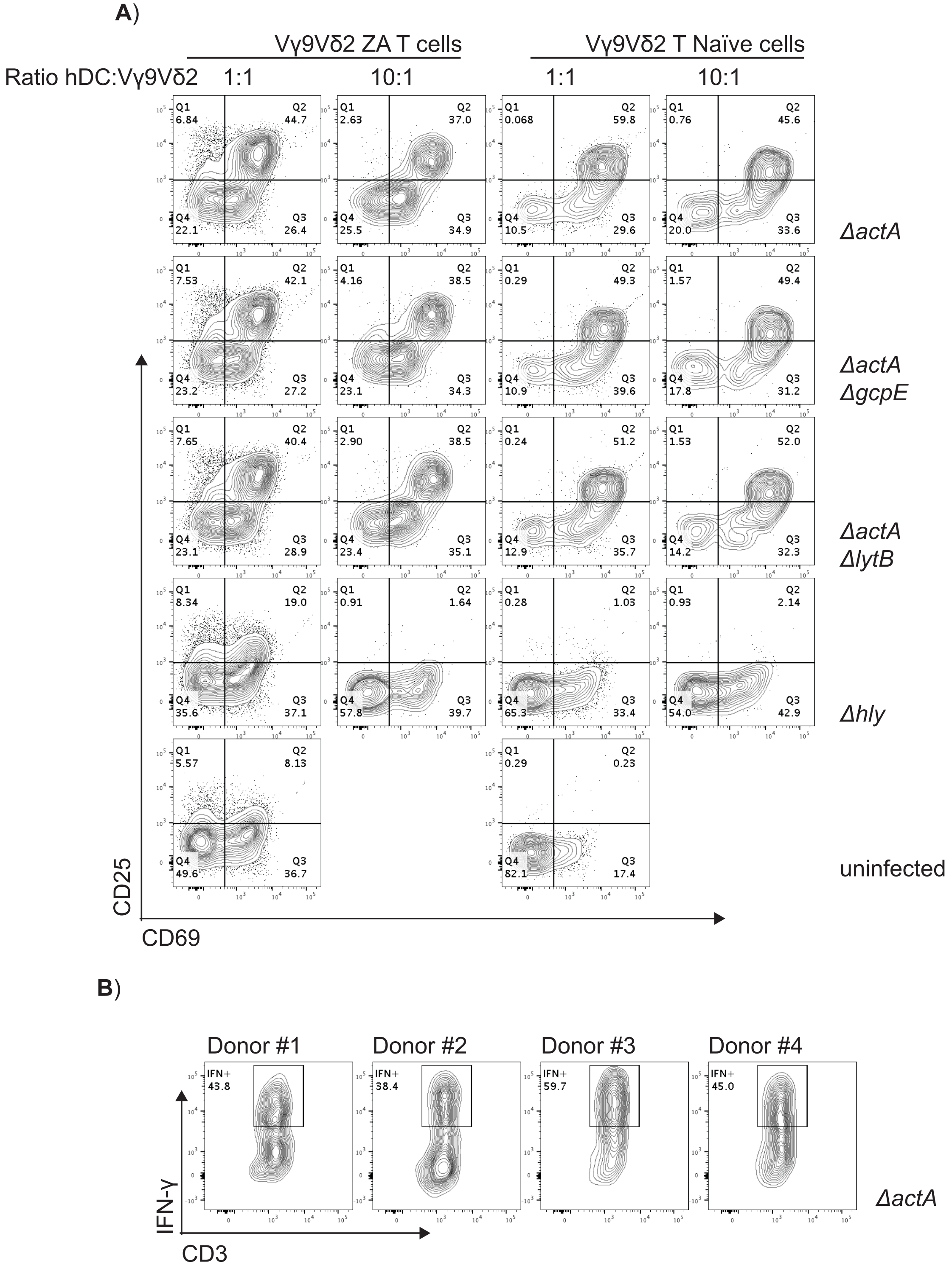


Suppl Figure 4. **A**) Activation of naïve Vγ9Vδ2 T cells. *Lm*-infected or uninfected Mo-DC were treated as described and incubated in the presence of various concentrations of

$V\gamma9V\delta2$ T cells before staining for the indicated markers. Flow-cytometry plots of $CD25^+$ $CD69^+$ $\delta2^+$ $CD3^+$ cells are representative results of at least 3 independent experiments. ZA: zoledronic acid expanded $V\gamma9V\delta2$ T cells. **B**) Activation of $V\gamma9V\delta2$ T cells obtained from various healthy donors. $V\gamma9V\delta2$ T cells were expanded from PBMCs obtained from different donors as described. Mo-DC were also obtained from each donor, infected with Lm, washed, incubated with the corresponding $V\gamma9V\delta2$ T cells and intracellular stained as described. Flow-cytometry plots of $IFN\gamma^+$ $\delta2^+$ $CD3^+$ cells are representative results of at least 2 independent experiments with 3 replicates each.

Suppl Fig 5

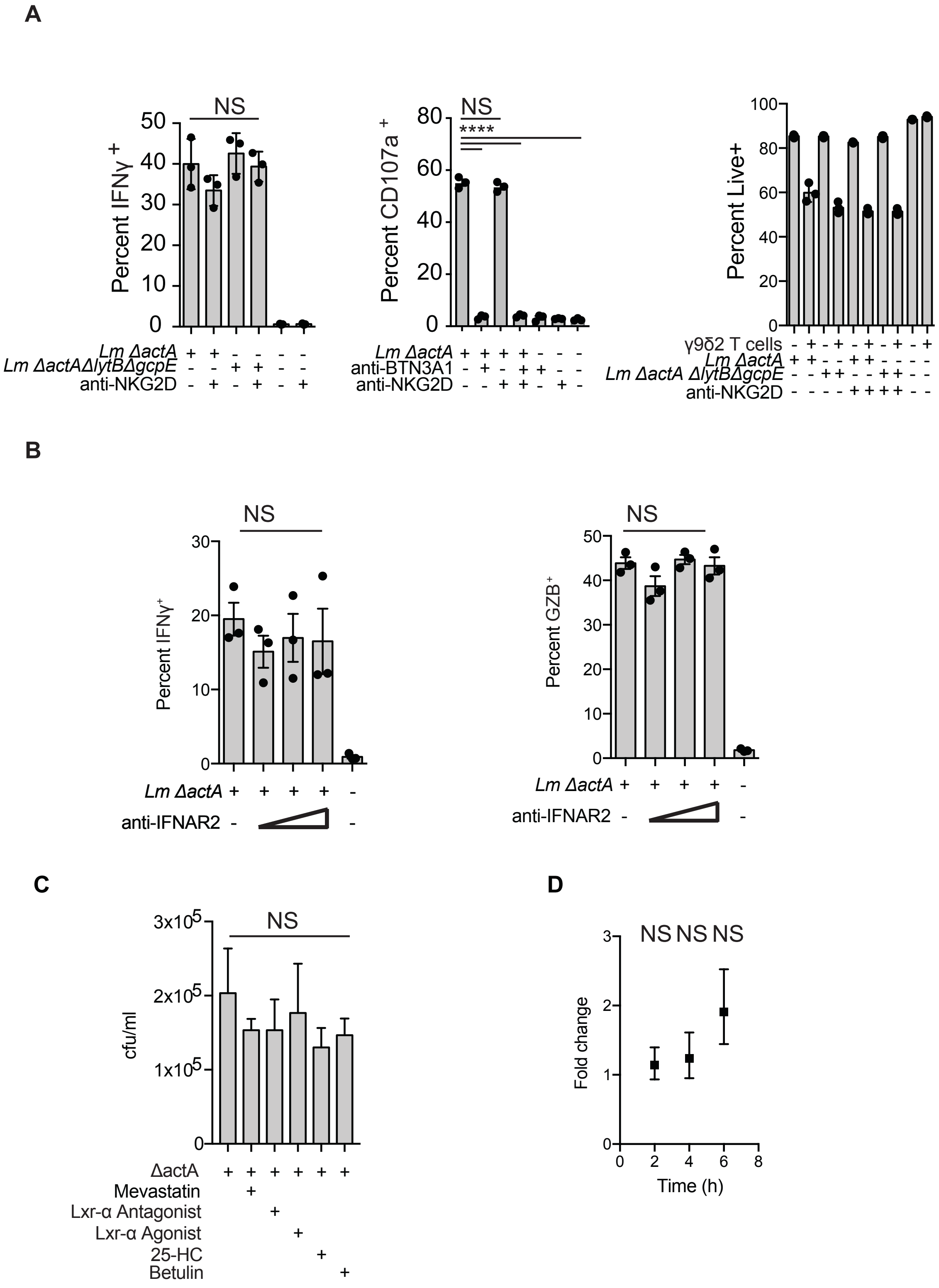


Suppl Figure 5. **A**) Activation of Vγ9Vδ2 T cells in the presence of anti-NKG2D blocking antibody. Left and center panels, quantitation of percent IFNγ⁺ δ2⁺ CD3 and CD107a⁺

$\delta 2^+$ CD3. *Lm*-infected Mo-DC were incubated with ex-vivo expanded V$\gamma$9V$\delta$2 T cells that were pre-incubated with anti-NKG2D blocking antibody or left untreated. When anti-BTN3A1 blocking antibody was used, Lm-infected or uninfected Mo-DC were incubated with this antibody previously to the addition of V$\gamma$9V$\delta$2 T cells. Right panel, quantitation of percent CD11c$^+$ Live$^+$. Cells were processed as above and stained for viability. **B**) Activation of V$\gamma$9V$\delta$2 T cells in the presence of anti-IFNAR2 blocking antibody. Left panel, quantitation of percent IFN$\gamma^+$ $\delta 2^+$ CD3$^+$; Right panel, quantitation of percent GZB$^+$ $\delta 2^+$ CD3$^+$. **C**) Determination of viable counts in the presence of various compounds. Mo-DC were pre-incubated with the indicated compound and infected at MOI=10 with *Lm*, cells were washed and further incubated for 3 h (4 hours post infection). Cfu was determined as described. **D**) *Lxr-$\alpha$* gene expression changes during *Lm* infection of Mo-DC. Data represents the mean ± SEM of each group. Statistics calculated by unpaired *t* test (**D**) or one-way ANOVA with Tukey's correction (**A**, **B** and **C**); ns = no significant differences observed between the groups analyzed.

**Supplementary Table 1**

| Primers name | Sequence 5' to 3' |
|---|---|
| ABCA1 F | ACATCCTGAAGCCAATCCTGA |
| ABCA1 R | CTCCTGTCGCATGTCACTCC |
| CH25H F | CTCTACCAGCATGTGATGTTTGT |
| CH25H R | CATGTCGAAGAGTAGCAGGCA |
| DHCR24 F | CACTGTCTCACTACGTGTCGG |
| DHCR24 R | CCAGCCAATGGAGGTCAGC |
| HMGCS1 F | CTCTTGGGATGGACGGTATGC |
| HMGCS1 R | GCTCCAACTCCACCTGTAGG |
| LSS F | GCACTGGACGGGTGATTATGG |
| LSS R | TCTCTTCTCTGTATCCGGCTG |
| LXRA F | ACACCTACATGCGTCGCAAG |
| LXRA R | GACGAGCTTCTCGATCATGCC |
| GAPDH F | CATGTTCGTCATGGGGTGAACCA |
| GAPDH R | AGTGATGGCATGGACTGTGGTCAT |
| IFNB1 F | ACAGCATCTGCTGGTTGAAG |
| IFNB1 R | GTCAGAGTGGAAATCCTAAG |
| APOAI F | CTCAAAGACAGCGGCAGAGACTA |
| APOAI R | ATCTCCTCCTGCCACTTCTTCTG |
| gcpE1 | GGTCGACCCTATCTCGATTGATCAAGATGAAC |
| gcpE2 | GGATCCGCTAGTTCAGGTGTCATAACTCGC |
| gcpE3 | CGATTATCTAAGGCTTTCCAAATCTTGGACTGGGCGAGTGT TTTCGCG |
| gcpE4 | CGCGAAAACACTCGCCCAGTCCAAGATTTGGAAAGCCTTA GATAATCG |
| lytB1 | GGTCGACAAGCCCATATCAAGTGTCATGTCTG |
| lytB2 | GGATCCCAGAATATACAGAGTGCACCTTCC |
| lytB3 | GGGTAGAATACGCTCAAAGTCCTGAGCAATCACCATCGCA TCGATGACTCC |
| lytB4 | GGAGTCATCGATGCGATGGTGATTGCTCAGGACTTTGAGC GTATTCTACCC |
| BTN3A1 F | GCTCTGAGTGCTGTGTTATGA |
| BTN3A1 R | CCTGCCTCAACTTCCATACTT |

Supplementary Table 1. Primers used in the manuscript.
